# A systematic review of interventions to increase awareness of mental health and well-being in athletes, coaches and officials

**DOI:** 10.1186/s13643-017-0568-6

**Published:** 2017-08-31

**Authors:** Gavin Breslin, Stephen Shannon, Tandy Haughey, Paul Donnelly, Gerard Leavey

**Affiliations:** 1 Sport and Exercise Sciences Research Institute, Ulster University Jordanstown Campus, Shore Road, Newtownabbey, BT370QB Northern Ireland; 2 The Bamford Centre for Mental Health and Wellbeing, Ulster University, Magee Campus, Derry, BT487JL Northern Ireland; 3 Sport Northern Ireland, House Of Sport, Upper Malone Road, Belfast, BT9 5LA Northern Ireland

**Keywords:** Mental health literacy, Sport, Resilience, Athletes, Interventions, Health promotion

## Abstract

**Background:**

The aim of the current study was to conduct a systematic review determining the effect of sport-specific mental health awareness programs to improve mental health knowledge and help-seeking among sports coaches, athletes and officials. The second aim was to review the study quality and to report on the validity of measures that were used to determine the effectiveness of programs.

**Methods:**

Sport-specific mental health awareness programs adopting an experimental or quasi-experimental design were included for synthesis. Six electronic databases were searched: PsycINFO, MEDLINE (OVID interface), Scopus, Cochrane, CINAHL and SPORTDiscus. Each database was searched from its year of inception to October 2016. Risk of bias was assessed using the Cochrane and QATSQ tools.

**Results:**

Ten studies were included from the 1216 studies retrieved: four comprising coaches or service providers, one with officials, four with athletes, and one involved a combination of coaches and athletes. A range of outcomes was used to assess indices of mental health awareness and well-being. Mental health referral efficacy was improved in six studies, while three reported an increase in knowledge about mental health disorders. However, seven studies did not report effect sizes for their outcomes, limiting clinically meaningful interpretations. Furthermore, there was substantial heterogeneity and limited validity in the outcome measures of mental health knowledge and referral efficacy. Seven studies demonstrated a high risk of bias.

**Conclusions:**

Further, well-designed controlled intervention studies are required. Researchers, practitioners and policy makers should adhere to available methodological guidance and apply the psychological theory of behaviour change when developing and evaluating complex interventions.

**Trial registration:**

PROSPERO CRD42016040178

**Electronic supplementary material:**

The online version of this article (10.1186/s13643-017-0568-6) contains supplementary material, which is available to authorized users.

## Background

Mental health is defined as ‘a state of well-being in which every individual realizes his or her own potential, can cope with the normal stresses of life, can work productively and fruitfully, and is able to make a contribution to her or his community’ [1]. Mental health problems are one of the main causes of disease burden, with major depression being the second leading cause of disability and a contributor to suicide and heart disease worldwide [2]. Globally, an estimated 350 million people are affected by depression, 60 million, by bipolar affective disorder, and 21 million, by schizophrenia/other psychoses [3]. In the USA, 20% of adults experience a mental health problem annually (30% among 18–25-year-olds) [4]. Similar figures have been reported in Europe and Australia [1]. There is relatively scant research on the mental health of athletes [5], and the prevalence of diagnosable psychiatric disorders in athletes is unclear [5–8]. However, elite athletes are just as likely as non-athletes to experience anxiety [5] or depression [9, 10].

Sports participation, particularly through physical activity, has many benefits to health and well-being [11]. Recent evidence suggests that competitive sport may contribute to poor mental health [12] and that athletes have specific risk factors for poor mental health [13]. These pressures described by Evans, Weinberg and Jackson [14] include; extended times being separated from family [15], negative emotional consequences of injury [16], increased risk of substance and alcohol abuse [17], worries that media outlets, or large populations, will be critical of them if they fail [12] and relationship problems [13]. Common life concerns for athletes include interpersonal conflict, employment qualms and financial difficulties. These pressures combined with how athletes interpret their sports performance failures can be problematic. For example, attribution styles (i.e., explanations for success or failure) have been linked to levels of emotional well-being, which in turn can contribute in part to depression [8].

Traditionally, athletes have been poorly supported to manage their mental health. Instead, sports culture celebrates mental toughness and disapproval of weakness disclosure [12]. Consequently, emotional and psychiatric problems remain hidden [18], a stigma that prevents athletes from seeking timely and appropriate help [12]. Furthermore, coaches also experience specific stressors and in some cases, may require better emotional support [19, 20] within a culture of performance and competition. Sports officials (referees, umpires, judges) also experience stress and burnout [21, 22]. Rainey [23] described four reasons for stress and burnout: fear of physical harm, fear of failure, time pressure and interpersonal conflict, with few mental health interventions on sports officials available and research at an exploratory stage [24].

Mental health literacy is the knowledge and beliefs about mental health disorders which aid recognition, management or prevention [25]. Most mental health information is perceived by the public as confusing [26] which presents a problem in raising awareness of the importance of caring for mental health. Despite evidence-based interventions being universally available to enhance mental health [27], few programs have been adapted for coaches, athletes and sport club settings. Coaches, who manage stress well, may be better equipped to prevent or deal with athletes’ stress more effectively [28]. Thus, training coaches in mental health awareness and mental health literacy may then improve the emotional climate of the performance environment enhancing interactions and positive relationships between an athlete and a coach [29].

Few evidence-based mental health awareness programs are designed for sports-specific populations despite the demand for context-specific programs [30]. However, many athletes, despite the access to relevant information and advice, do not seek help, as the disadvantages of doing so are perceived to outweigh the benefits [12]. The disadvantages included the loss of playing time, being perceived as mentally weak or lacking commitment [31]. Moreover, the fear of disclosure may damage their athletic career plans. Additional barriers for young people seeking help are poor mental health literacy and stigma, while the main facilitators of positive help-seeking are; having a current positive relationship with a health professional or doctor, an emotional intelligence and/or a positive social support [9].

It has been reported that gender is often a determinant of help-seeking [32], wherein male’s adoption of masculine norms such as strength and stoicism negatively impacts upon their willingness to seek help and reinforces maladaptive coping styles [33]. Furthermore, recent evidence suggests that depressive symptoms (e.g. mood disorders, substance abuse) are moderated by gender [34]. In support of this perspective, a study of female athletes’ exhibited 1.844 times the risk for developing clinically relevant symptoms compared to male athletes [35].

Mental health awareness programs offer the opportunity to enhance help-seeking behaviours and improve well-being among athletes through counselling or psychological skills training (see [36] for an example) which disseminate knowledge related to mental health conditions and treatment [37]. A recent systematic review [5] on the incidence and nature of mental health and well-being of elite athletes concluded that few studies were well reported (25%) and lacked methodological rigour and there was a paucity of interventions. However, the review focused on elite-athletes only, and potential interventions for non-elite athletes in club settings for coaches and referees remains to be reviewed. Given the recent interest in the topic evidenced in part by the recent special issue in Frontiers in Psychology consisting of ten articles called “Mental health challenges in elite sport: balancing risk with reward”, a review of available interventions is required. Also, determining what interventions work is timely as in the USA college athletic departments, the USA Olympic Committee, USA Olympic Governing Bodies and professional sports organisations now provide athletes, coaches and support staff with access to mental health professionals. The National Association of Athletic Trainers has published a consensus statement that addresses the growing concern for mental health issues in athletes and provides recommendations for development in this area. The National Collegiate Athletic Association [38] published *Mind*, *Body and Sport* identifying the need for college athletic departments to address this growing concern and provide guidelines for implementing a dedicated response to assist student athletes [12].

Similarly, in March 2015, the UK government presented a ‘mental health in sport’ initiative. Several sports associations, including the Rugby Football Union, UK Athletics, British Swimming, the England and Wales Cricket Board and the Football Association, signed a contract pledging to support the elimination of stigma, narrow-mindedness and prejudice surrounding mental health. This was a stride forward for the UK concerning the de-stigmatisation of mental health and an encouraging act toward facilitating help-seeking. Furthermore, the Sport and Recreation Alliance in the UK launched the Mental Health Charter for Sport and Recreation to take positive steps to address and encourage people to be open about mental health issues. Within Northern Ireland, Sport Northern Ireland (SNI) has reviewed mental health awareness programs nationally in the development of a new strategy for those involved in sport [39]. The European Federation of Sport Psychology is in the process of developing a position statement related to the mental health of elite athletes [7] whilst in Australia, elite athlete Brief Counselling Support programs have been put in place as well as mental health awareness programs for athletes.

The aim of the current study was to conduct the first systematic review determining the effect of sport-specific mental health awareness programs to improve mental health knowledge and help-seeking among coaches, athletes and officials. The second aim was to review the study quality and to report on the validity of measures that were used to determine the effectiveness of programs. A description of intervention programs delivered will be provided and recommendations for those in the process of designing and evaluating mental health programs for athletes, coaches and officials are proposed.

## Methods

### Protocol

All methods of data analysis and reporting followed the Preferred Reporting Items for Systematic Reviews and Meta-Analyses (PRISMA) guidelines [40]. A protocol is available on the PROSPERO (an international database of prospectively registered systematic reviews in health and social care) database and can be accessed online (registration number: CRD42016040178). A checklist is provided as Additional file 1.

### Eligibility criteria

#### Types of studies

Randomised or clustered randomised controlled trials and quasi-experimental studies that did not use a prespecified randomisation processes when selecting the treatment and comparator condition [41] were included. Studies comparing the treatment with a comparison group, more than one intervention group, or within subjects across time (i.e. pre-post testing) were included. Studies were required to have been published in the English language. The decision was taken to restrict our inclusion criteria to only peer-reviewed literature as the grey literature (e.g. dissertations, reports, policy documents) is heterogeneous and little methodological guidance exists for the systematic retrieval, analyses and reproducibility of such work [42].

#### Types of participants

Participants were children, adolescents or adults who are considered as an athlete, leader, coach, official or member (e.g. service provider) within a professional or amateur sporting club or organisation.

#### Types of interventions

Mental health interventions that take a general approach to improving awareness of mental health or interventions tailored to focus on a specific mental health disorder (e.g. depression, anxiety, substance misuse). While eating disorders are a relevant topic for mental health awareness programs, we decided to exclude these studies because a recent systematic review focused on eating disorder prevention initiatives for athletes (*n* = 11, see [6]).The mode of delivery was individual, group or web-based. To be eligible for inclusion, interventions had to take place within a sports setting (i.e. sports club, sports environment). As many definitions of sport exist, we applied Rejeski and Brawley’s [43] definition for consistency: a structured physical activity that is competitive, rule-governed, and characterised by strategy, prowess and chance. Exclusion criteria were applied to interventions that were considered as being outside the domain of sport (e.g. physical activity, exercise, leisure, art and music).

#### Types of outcome measures

Studies needed to include at least one outcome measure which we categorised as related to mental health attitudes (i.e. *stigma*, prejudice), knowledge of mental health (i.e. disorder and symptom recognition) or behaviour regarding mental health (intended or actual help-seeking), mental health competencies (i.e. mindfulness, coping) or specific mental health (i.e. anxiety, depressive symptoms, positive affect) and well-being (i.e. subjective/psychological well-being domains, life satisfaction) outcomes. Only quantitative studies were included as it would be difficult to assume a level of generalisability between quantitative and qualitative outcomes. Furthermore, a qualitative review could be reported as a separate article.

### Information sources and search strategy

We used electronic databases and also manually checked reference lists of articles. Six electronic databases were searched: PsycINFO, MEDLINE (OVID interface), Scopus, Cochrane, CINAHL and SPORTDiscus. Each database was searched from its year of inception to October 25, 2016. Search terms used keywords, truncation and MeSH terms as appropriate for each database’s indexing reference [44]. The search was stratified into four categories: sport, participants, setting and method of treatment. Search terms were chosen based on previous research, theory and practice (see Table 1). The first category used sport as a single term as a sport is central to the objective of the review. As with previous systematic reviews in sport [45], the second category used descriptors that are associated with participation or membership within the sport. The third category depicted broadly cited sports settings in sports development literature [46] and also included Internet-based terms to account for recent developments of online mental health interventions [47]. Lastly, search terms in the fourth category were applicable terms to constructs associated with mental health and well-being [48], mental health knowledge [49] and coping strategies appropriate for mental health interventions [50]. A full electronic search of the PsycINFO search is uploaded as an Additional file 2.

**Table 1:** Search terms used in PsycINFO search reflecting keywords, MeSH terms and suffixes

Category	Key terms
Sport	Sport$
Participants	Leader$ or athlete$ or teacher$ or instructor$ or player$ or member$ or participant$ or coach$
Setting	Sport adj3 (organi#ation$ or club$ or governing bod$ or cent$ or school$ or setting$ or internet or online or website$ or web site$ or web based)
Method of treatment	mental$ adj3 (health or wellbeing or well being or well-being or wellness or ill$) or anxiety or depress$
Limiters	English language and peer reviewed

*$* search singular or plural, *adj3* adjacent, *#* replaces one character

### Study selection and data collection process

Study selection was completed in three phases. First, database searches were exported to RefWorks software into a master folder. All titles and abstracts were screened by one researcher. Duplicates were removed, and all abstracts were exported to a subfolder (i.e. included for follow-up or excluded). All relevant abstracts were printed and screened by a second researcher to assess their eligibility for full-text printing and screening. Second, to ensure inter-rater reliability, two researchers independently screened 10% of all excluded titles and abstracts. Although a high level of agreement (> 95%) was reached, two potentially relevant abstracts were highlighted and subsequently screened by two authors using the inclusion criteria. They were found to be irrelevant and were excluded. Third, full-text eligibility assessment was performed independently in an un-blinded standardised manner by two researchers (SS and GB) using the screening tool (see Table 2). The remaining included articles were divided between two researchers, and all predefined data (see below) were extracted by one researcher and cross-verified by a second for the synthesis of results.

**Table 2:** Screening tool for independent author screening

	Yes	No	Comments
Language Is the full paper in English?			
	Go to next question	Exclude	
Peer review Has the paper been peer reviewed?			
		Exclude	
Type of study Is the study described as one of the following:			
i. Clustered randomised controlled trial			
ii. Non-randomised controlled trial/quasi-experimental study			
iii. Pre/post-test study design			
		Exclude	
Participants Are the participants’ children, adolescents or adults who are considered as an athlete, leader, coach or member within a sporting (amateur or professional) organisation?			
		Exclude	
Intervention type Does the intervention contain a mental health and/or well-being training component?			
		Exclude	
Intervention location Is the intervention within a sport setting (sport: ‘rule-governed, structured, competitive gross movement characterised by physical strategy, prowess and chance’ (Rejeski and Brawley [37]). Exclude if intervention is outside the domain of sport (i.e. leisure, exercise, art, music).			
		Exclude	
Outcomes i. Does the study report mental health AND awareness, knowledge, first aiding, fitness, intentions, action planning, self-efficacy/competence? ii. Does the study report mental health outcomes (i.e. anxiety, depression, or subjective well-being markers).			
			
Include for follow-up			

### Data items, summary measures, synthesis and analysis of results

Detailed descriptive information from each intervention including the author(s) and year of study; study design features (e.g. data collection points, inclusion of a control group or not); sample characteristics including age and gender; mode of delivery; mental health descriptor (i.e. increase knowledge, improve attitudes or reduce depressive symptoms) (see, Table 3). For assessing the effect of the interventions, we obtained the name of the outcome measure(s), reported value(s) for intervention effectiveness (i.e. *p* value, effect size) and based on prior research [51] that provided a narrative commentary on study design methods that may influence the generalisability of study effects. As all of the outcomes measured derived from scales, we observed statistically significant quantitative effects on the basis of *p* < .05 [52], and a small, medium or large effect size as *d* = .2, .5 or .8, respectively [53]. We reported the effects of each study in Table 4. For combining and reporting the results, we inspected each study’s outcomes and categorised them in accordance with the following key mental health constructs [5]: stigma, mental health knowledge, referral efficacy/confidence, help-seeking intentions and behaviour, well-being, and additional outcomes. A meta-analyses were not conducted as substantial heterogeneity was found for construct measurement and operationalisation (e.g. intentions to help other with a mental health problem vs. intentions to help oneself), and many studies did not report statistical tests for significance. No additional subgroup or sensitivity analyses were conducted, as these were not in line with our study aims.

**Table 3:** Descriptive information for the ten included studies

Authors (year of study)	Study design; duration	Sample characteristics	Mental health descriptor; mode of delivery
Bapat, Jorm, and Lawerence [67]	Pre-post design; 3 weeks	Sport club leaders (*n* = 40; age = 38.62; 16 males, 24 females)	Mental health literacy through mental health first aid training; 8 h training program delivered over 3 sessions using a range of presentations, tasks and homework.
Breslin, Haughey, Donnelly, Kearney, and Prentice [37]	Controlled trial; 1 day (3 h session)	Sport coaches (*n* = 244; 126 males, 118 females)	Mental health awareness program involving videos and discussions with athletes who have experienced depression; 3 h program delivered in one session by a public health agency provider
Donohue et al. [68]	Single subject pre-post and follow up design; 4 months	Athletes with previous history of substance abuse or dependence (*n* = 7; age = 20; 4 males, 3 females)	Modifying behavioural and cognitive skills to overcome substance abuse; 12 individual meetings on a range of topics
Gulliver et al. [69]	Randomised control trial; 5 weeks	Elite athletes (*n* = 59; age = 25.5; 16 males, 43 females)	Mental health literacy; participants were allocated to one of a series of online psycho-educational programs
Pierce, Liaw, Dobell and Anderson [71]	Pre-post design (club leaders); controlled trial (football players); 3 weeks	Club leaders (*n* = 36; age = 45); and football players (*n* = 275; age = 21)	Mental health literacy; 12 h psycho-educational group sessions for leaders; information sessions were conducted with players alongside informal information
Longshore and Sachs [70]	Controlled trial; 6 weeks	College coaches (*n* = 20; age = 34.5; 8 males, 12 females)	Mindfulness training program to develop emotional awareness and reduce stress; an initial 1.5 h group session followed by a 6 week home program
Sebbens, Hassmen, Crisp and Wensley [29]	Controlled trial; 1 day (4 h)	Coaches, trainers, support staff and service provides (*n* = 166; age = 37.8; 83 males, 83 females)	Mental health knowledge and confidence program; 4 h applied workshop involving case studies, role-playing and videos
Slack, Maynard, Butt and Olusiga [72]	Single subject pre-post design; 1 season (approximately 6 months)	Referees (*n* = 3; age = 28.67; 3 males)	Mental toughness education and training program; six monthly workshops involving four individual-based and two group-based sessions consisting of role-playing and cognitive behavioural therapy techniques
Tester, Watkins and Rouse [73]	Pre-post design; 2 school years	‘At risk’ schoolchildren enrolled in a sports program (*n* = 991)	Preparation for life skills (i.e. pro social behaviours, stress management) were taught by sporting mentors through a basketball program in and outside classroom settings over the course of 2 years
Van Raalte, Cornelius, Andrews, Diehl and Brewer [74]	Randomised controlled trial; 1 day (online session lasted at least 10 min)	Student athletes (*n* = 153; age = 19.63; 46 males, 103 females)	Mental health literacy; web-based program using exercises and interactive material

**Table 4:** Study outcome measures, main findings and comments on study

Authors (year of study)	Mental health outcome measure(s)	Main findings	Comments
Bapat, Jorm and Lawerence [67]	SQ KQ ?V	Significant reduction in levels of stigma (*p* < .001); increase in knowledge about mental disorders (*p* < .01); increased confidence to help someone with mental disorder (*p* < .001)	Small sample size (*n* = 40); no control group; no effect sizes reported; no follow-up data
Breslin et al. [37]	RIBS MAKS ?3	Significant improvement for intervention group in comparison to control on mental health knowledge, confidence in ability to help someone, and intention to offer help to individuals with a mental health problem (all findings *p* < .001)	No randomisation method; no follow-up data; no effect sizes reported; intended behaviour was reported rather than actual behaviour
Donohue et al. [68]	SCL-90-R BDI SARI TLFB RAB	Psychiatric functioning mean scores improved from baseline to post. Improved scores remained stable at 1- and 3-month follow-up; depressive mean scores decreased from baseline to post-intervention and remained stable at follow-up. Improvements were shown for all relationship domains	Small sample size (*n* = 7); no values provided for study effects (i.e. *p* value or effect); no control group
Gulliver et al. [69]	ATSPPH-SF GHSQ AHSQ D-Lit A-Lit DSS GASS	No significant interaction effect for help-seeking attitudes, intentions or behaviour from baseline to follow-up. However, significant positive interaction effects were observed for depression (*p* < .05) and anxiety literacy (*p* < .01), and anxiety stigma (*p* < .05) from baseline to follow up relative to control group	Effect sizes for the significant positive interaction effects differed for treatment condition (literacy condition, feedback condition and help-seeking) in comparison to control, ranging from small to medium to large. Caution is advised when interpreting findings as the sample size was small
Pierce, et al. [71]	?1 ?2	Leaders: Significant positive change in recognition of mental illness (*p* < .001), confidence that anti-depressant medication can be helpful (*p* < .01) and confidence in helping someone with mental health problem (*p* < .001). Players: no significant changes	Leaders: Small sample size (*n* = 36), no control group. Players: Unclear information on their attendance and involvement in the intervention. No effect sizes reported
Longshore and Sachs [70]	MAAS TMS STAI PANAS BRUMS	No significant interaction effect reported for anxiety, mindfulness awareness or experience, or moods. A significant interaction effect was reported for a reduction in negative affect (*p* < .05, ES = .21)	Small sample size (*n* = 20). Despite largely non-significant results, mean scores showed positive trends, and effect sizes were generally small to moderate. Interviews with participants showed positive changes in coaches’ personal life and mindfulness
Sebbens, et al. [29]	D-Lit A-Lit ?3	A significant interaction effect was recorded for the intervention group in comparison to control on depression and anxiety literacy and confidence to help at time 2 (2 weeks post-intervention) (*p* < .001) but not at time 3 (4 weeks post-intervention)	No randomisation method; no effect sizes reported; intended behaviour was reported rather than actual behaviour
Slack, et al. [72]	SGMT RSMT	Positive mean score changes were recorded for all three referees’ general and referee-specific mental toughness scores in the intervention phase in comparison to baseline	No values provided for study effects (i.e. *p* value); no control group; qualitative data strengthened the evaluation of program; referees’ performance increased
Tester, Watkins and Rouse [73]	SCQ	Overall mean improvement of 44% (6–11-year olds) and 18% (12–16-year olds) in post-test scores in comparison to baseline for self-concept	No values provided for study effects (i.e. *p* value, effect size); no control group
Van Raalte, et al. [74]	MHRES MHRK	Significant positive changes were observed for mental health referral efficacy (*p* < .001, ES = 0.1) and knowledge (*p* < .01, ES = .04) for the intervention group in comparison to control group	Intervention was tailored for the population. Qualitative data showed positive feedback for intervention acceptability
Summary	Substantial heterogeneity in measures used to assess mental health knowledge (*n* = 4) and help-seeking intentions (*n* = 4)	Positive significant findings for all outcomes measured (*n* = 2); positive significant findings on at least one outcome measure (*n* = 7). Non-significant findings (*n* = 2). No statistical tests for significance (*n* = 3). Actual behaviour change for help-seeking (*n* = 0)	No control group (*n* = 5); small sample size (*n* = 4); randomisation (*n* = 2)

*SQ* Stigma questionnaire, *KQ* Knowledge questionnaire, *?V* no name given to confidence measure for vignette, *SCL-90-R* Global Severity Index of the General Psychiatric Symptoms-90-Revised, *BDI* Beck Depression Inventory, *SARI* student-athlete relationship instrument, *TLFB* timeline follow back, *RAB* risk assessment battery, *ATSPPH-SF* Attitudes Toward Seeking Professional Psychological Help-Short Form, *GHSQ* help-seeking intentions, *AHSQ* actual help-seeking, *D-Lit* Depression Literacy Questionnaire, *A-Lit* Anxiety Literacy Questionnaire, *DSS* Depression Stigma Scale, *GASS* Generalised Anxiety Stigma Scale, *?1* no name given to measure with questions around mental health recognition, knowledge and confidence, *?2* no name given to customised measure around attitudes and recognition of depression in clinical scenario; *MAAS* Mindful Attention Awareness Scale, *TMS* Toronto Mindfulness Scale, *STAI* State and Trait Anxiety Inventory, *PANAS* Positive and Negative Affect Schedule, *BRUMS* Brunel Mood Scale, *MHRES* Mental Health Referral Efficacy Scale, *MHRK* Mental Health Referral Knowledge Scale, *RIBS* Reported and Intended Behaviour Scale, *MAKS* Mental Health Knowledge Scale, *?3* no name given to measure with questions around mental health confidence to help, *SGMT* sport-general mental toughness, *RSMT* referee-specific mental toughness, *SCQ* Song And Hattie Self-Concept Questionnaire

### Risk of bias within and across studies

For profiling the study quality and risk of bias, we adopted the principles of the Cochrane Collaboration for assessing methodological quality in systematic reviews [41]. As included studies were either categorised as randomised or non-randomised designs, each study’s design was matched with an applicable assessment of bias tool. For randomised controlled trials, we used the Cochrane Collaboration’s tool for assessing the risk of bias [54]. The tool includes six domains of bias such as selection, detection and reporting bias. Each domain is coded as high, low or unclear for the relative risk of bias and an overall judgement is accumulated. For non-randomised studies, we used the Quality Assessment Tool for Quantitative Studies (QATSQ) [55] which is recommended for use in systematic reviews [56]. The QATSQ tool is scored based on six domains of bias including selection bias, confounding bias and withdrawals and dropouts. Based on the predefined bias criteria, the domains were scored as either weak (3), moderate (2) or strong (1). Studies with no weak ratings and at least four strong were considered strong, while studies with fewer than four strong ratings and one weak rating were considered moderate, and studies with two or more weak ratings were considered weak [55].

Based on the Cochrane Collaboration’s recommendations [54], we reported on the risk of bias across studies by summarising the cumulative bias for each outcome in the Cochrane and QATSQ tools. To facilitate reporting of bias across the studies, additional rows and columns were added to the tools.

Outcome measures were also assessed for validity as they can influence the generalisability of study findings [52]. The study adapted criteria used in a recent systematic review of mental health interventions [47]. Scales were considered acceptable if they met one or more of the following: a Cronbach’s alpha value of above .7, reporting of acceptable goodness of fit indices using confirmatory factor analysis [57], test-retest, construct or concurrent validity assessments or the authors referenced a previous study that validated the scales through the above methods.

## Results

A total of 1216 titles and abstracts were reviewed (242 from PsycINFO; 39 from MEDLINE; 153 from Scopus; 128 from Cochrane; 381 from CINAHL; 273 from SPORTDiscus). One further article was identified from one of the author’s knowledge of an article accepted and in press. After removal of duplicates (*n* = 88), 1129 titles and abstracts remained. Of these, 1023 were identified as irrelevant and were excluded. Ten percent of excluded titles and abstracts were screened by two researchers, and a consensus was reached for their exclusion. A total of 106 articles were identified as relevant and underwent a further detailed screening for full-text printing eligibility; of these, 20 met the criteria for a standardised independent full-text screening by two authors.

**Fig. 1:**
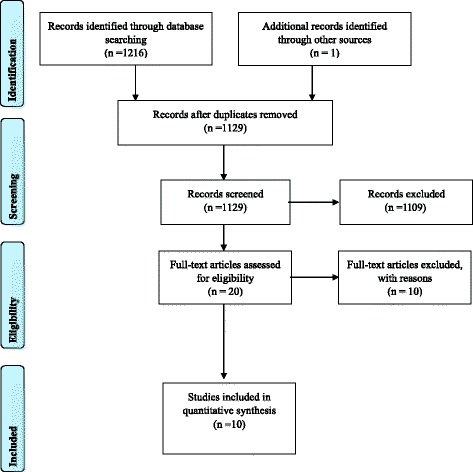
PRISMA 2009 flow diagram

From the 20 articles, authors agreed upon ten articles to be excluded because they did not meet the inclusion criteria on at least one level. One article was a review [58], others were a cohort study [59], diary study [60], drug-testing study [61], a muscular relaxation program [62], a physical injury prevention intervention [63], and two others were a description of a mental health charity or included no mental health component. Three articles [64–66] were based on eating disorders and, as discussed above, were subsequently excluded on the basis of a recent systematic review focusing on this topic [6]. Of the remaining ten articles [29, 37, 67–74], there was 100% author agreement for their inclusion for further review synthesis (see Fig. 1). A further, 12 references were identified by hand-searching the reference lists of the ten included articles. However, none of these articles met the inclusion criteria for the review, they were either chapters in books, conference abstracts, a statement on mental health awareness, a cross-sectional survey or reported qualitative findings.

### Study characteristics

Study characteristics are detailed in Table 3. Across the ten studies, 1994 participants took part, which included 302 males and 386 females. Two studies did not report gender, 995 [73] and 311 [71] participants, respectively. The interventions were delivered to a range of sports participants including athlete and elite athletes (*n* = 3), officials (*n* = 1) and ‘at-risk’ children (*n* = 1), with the most common being coaches and service providers (*n* = 5). Studies adapted various designs including intervention pre-post testing [67, 71–73], randomised control trial [69, 74], controlled trial [29, 37, 70] and a descriptive case trial [68]. The mode of delivery was mixed and ranged from a group setting (*n* = 6), individual counselling format (*n* = 1) and online (*n* = 2) to at home (*n* = 1).

### Study results

The name of the authors who conducted the study, the year, the design, study duration, sample characteristics, mental health descriptor employed, mode of delivery, mental health outcome measure(s), main findings and general comments regarding each study are summarised in Table 4. Studies selected for inclusion were published between November 1999 and December 2016/in press.

Five studies [29, 37, 69, 70, 74] included a control group, of which two [69, 74] used randomisation procedures. Sample sizes varied as five of the ten studies [67, 68, 70–72] had 40 participants or fewer. Seven studies [37, 67, 70–74] collected outcome measures pre- and post-intervention, while three studies [29, 68, 69] collected outcome measures at pre, post and follow up.

Findings from the studies were mainly in support of introducing knowledge-based mental health programs in sports settings (examples of interventions are described in Table 3).

Of the studies that included a follow-up, two [68, 69] maintained some of their effects, while one [29] did not. Three studies did not report a statistical test of significance [68, 72, 73], and a further seven [29, 37, 67, 68, 71–73] did not report effect sizes which limit clinically meaningful interpretations. The effects of the programs on each key construct are described below.

### Effects on stigma

Stigma around anxiety [69] and depression [67] was significantly reduced, with one study [69] having a null effect on depression stigma. The Breslin et al. study [37] reduced stigma surrounding socialising with others with a mental disorder.

### Effects on mental health knowledge

The five studies [29, 37, 67, 69, 71], reporting on mental health knowledge, all indicated a statistically significant positive increase in the participants’ ability to recognise a mental health disorder. A further study [74] reported an increase in mental health referral knowledge. However, all but two [69, 74] reported an effect size, limiting clinically significant interpretations. Just two studies [29, 69], used the same instruments to assess mental health knowledge, with substantial heterogeneity in the remaining four studies’ measurement and operationalisation of the mental health knowledge construct (see Table 4).

### Effects on referral efficacy/confidence to help someone with a mental health problem

Perceptions of self-efficacy to help someone with a mental health problem were enhanced in five studies [29, 37, 67, 71, 74]. However, all but one [74] measured referral efficacy with a validated scale, with the former four [37, 67, 71, 74] measuring referral efficacy using a single item that had not been previously validated.

### Effects on help-seeking intentions and behaviour

One study [37] reported an increase in intentions to offer help to those with a mental health problem, while another [69] indicated a null effect for intentions to seek help for oneself. Actual behaviour change was not achieved in any of the studies.

### Well-being and additional outcomes

Three studies [68, 70, 73] reported improvements in well-being outcomes, with one finding improvements in self-concept [73], one reducing depressive symptoms [68] and one decreasing negative affect [70]. Of these three studies, just one [70] reported statistical tests for significance (i.e. *p* value and effect size), limiting clinically relevant interpretations. Furthermore, this study [70] also reported null effects for anxiety, mood states and mindfulness awareness. Positive findings were reported for mental toughness [72], relationship domains [68] and substance abuse [68], but were not confirmed with statistical tests for significance.

### Risk-of-bias assessment

Risk-of-bias assessment for the two randomised studies is presented in Table 5. The two studies using randomisation methods demonstrated a low [69] and unclear [74] risk of bias. There was no high risk of bias scored for any of the domains across the two studies. Information was not provided on selection, performance and detection bias in [74], giving the design an overall judgement decision as unclear. Across the studies, bias was mixed for random sequence generation, allocation concealment and blinding of participants with [69] scoring low on those domains and [74] scoring unclear. Collectively, bias was unclear for blinding of outcome assessors, and both demonstrated a low risk of bias for (a) missing data, (b) selective reporting and (c) other biases.

**Table 5:** Risk of bias for randomised studies using Cochrane risk-of-bias tool

Study	Random sequence generation	Allocation concealment	Blinding of participants and personnel	Blinding of outcome assessment	Incomplete outcome data	Selective reporting	Other bias	Summary
Gulliver et al. [69]	^a^Automated computer system used	^a^Conditions allocated by researchers not involved in day-to-day management	^a^Described method used to reduce likelihood of participant knowledge of intervention	^b^Unclear whether assessors had knowledge of treatment groups when assessing effects	^a^Analyses adjusted for data being missing at random	^a^All outcome measure effects were reported, along with effect sizes for each group	^a^Study limitations were addressed and caution is urged when interpreting significant effects	Low risk of bias for this study. One domain (blinding of outcome assessors) was unclear but it is unlikely if that influenced the results given the online format of the intervention and data collection
Van Raalte et al. [74]	^b^Method not disclosed	^b^Unclear who performed randomisation	^b^Unclear if participants were or were not blinded to their intervention	^b^ Unclear whether assessors had knowledge of treatment groups when assessing effects	^a^ Analyses adjusted for data being missing at random	^a^All outcome measure effects were reported, along with effect sizes for each group	^a^Authors were transparent about each stage of the intervention design	Unclear risk of bias for this study. Information on selection, performance and detection bias was not disclosed, though attrition and reporting bias was low
Summary of bias across studies	Random sequence generation was performed in both studies, but one did not disclose the method	Methods of allocation were mixed, with one not providing information and the other having a low risk of bias	Across the two studies, one was unclear for blinding participants and the other controlled for contamination	Both studies demonstrated an unclear risk of bias for blinding the assessors’ knowledge	The risk of bias was low for both studies on controlling for missing data	There was a low risk of bias across the studies for reporting outcomes	Transparency was ensured by both studies, resulting in a low risk of bias	Findings were mixed for sequence generation, allocation concealment and blinding of participants, collectively unclear for blinding outcomes, and both positive in terms of controlling for missing data, selective reporting and other biases

^a^Low risk of bias

^b^Unclear risk of bias

^c^High risk of bias

Risk of bias for the eight non-randomised studies is presented in Table 6. Seven studies were found to have a weak study quality. One study [37] was found to be of moderate quality, scoring a mixture of moderate and strong on five domains, and weak on disclosing information on withdrawals and dropouts. Despite four studies scoring either strong [29, 70] or a mixture of moderate and strong [68, 73] on four domains, they also scored weak for at least two domains. The remaining three non-randomised studies all scored weak on at least three domains.

**Table 6:** Risk of bias for non-randomised studies using the QATSQ tool

Study	Selection bias	Study design	Confounders	Blinding	Data collection methods	Withdrawals and dropouts	Summary
Bapat, Jorm and Lawerence [67]	2 Participants are very likely to be representative Cannot tell the percentage of participants who agreed	2 Study is designated as a cohort analytic study	3 There were gender and age differences that may have influenced the outcomes between participants and these were not controlled for in analysis	3 Outcome assessors knew intervention status, and blinding of participants to research question is not described	3 The validity and reliability of the instruments are not described	3 Withdrawals and dropouts were not described	Weak quality: as this study scored four weak ratings, the overall judgement is weak quality
Breslin et al. [37]	2 Participants are very likely to be representative Cannot tell percentage of participants who agreed.	1 Study is designated as a controlled clinical trial	1 Confounders (gender, sport type) were similar across control and intervention groups	2 Cannot tell if outcome assessors were aware of intervention status and cannot tell if intervention participants were aware of research question	1 Tools were shown to be valid and reliable.	3 Cannot tell if there were withdrawals or dropouts	Moderate quality: As this study scored one weak rating the overall judgement is moderate quality
Donohue et al. [68]	1 Participants are very likely to be representative All participants agreed to participate	2 Study is designated as a cohort analytic study	3 There were gender, ethnic and age differences that may have influenced the direction of result. These were not controlled for in the analysis	3 Outcome assessors knew intervention status, and the participants knew intended outcome of the research (i.e. developing intervention)	1 The validity and reliability of the instruments is described	2 There was a 70% follow-up rate from those that consented and completed the intervention	Weak quality: as this study scored two weak ratings, the overall judgement is weak quality
Pierce, et al. [71]	2 Participants are very likely to be representative Cannot tell the percentage of participants who agreed	2 Study is designated as a cohort analytic study	3 There were age and education differences that may have influenced the direction of result these were not controlled for in the analysis	3 Outcome assessors knew intervention status, and the participants knew intended outcome of the research (i.e. respond to mental health problems)	3 The validity and reliability of the instruments is not described	2 There was a 66% follow-up rate from those that consented and completed the intervention	Weak quality: as this study scored three weak ratings, the overall judgement is weak quality
Longshore and Sachs [70]	1 Participants are very likely to be representative Above 80% of participants agreed to participate	1 Study is designated as a controlled clinical trial.	1 No significant differences were found between the groups before the intervention	3 Outcome assessors knew intervention status, and the participants knew intended outcome of the research (i.e. benefits of mindfulness)	3 The validity and reliability of the instruments is not described	1 There was a > 80% follow-up rate from those that consented and completed the intervention	Weak quality: as this study scored two weak ratings, the overall judgement is weak quality
Sebbens et al. [29]	1 Participants are very likely to be representative Above 80% of participants agreed to participate	1 Study is designated as a controlled clinical trial.	1 No significant demographic differences were found between the groups before the intervention	3 Outcome assessors knew intervention status, and blinding of participants to research question is not described	3 The validity and reliability of the instruments is not described	1 There was a > 80% follow-up rate from those that consented and completed the intervention	Weak quality: As this study scored two weak ratings, the overall judgement is weak quality
Slack et al. [72]	1 Participants are very likely to be representative Above 80% of participants agreed to participate	2 Study is designated as a cohort analytic study	3 Confounding variables were not discussed	3 Outcome assessors knew intervention status, and blinding of participants to research question is not described	3 While one measure was referenced as valid and reliable, no information was reported on validity and reliability of another measure (RSMT)	1 There was a > 80% follow-up rate from those that consented and completed the intervention	Weak quality: As this study scored three weak ratings, the overall judgement is weak quality
Tester, Watkins and Rouse [73]	2 Participants are very likely to be representative Cannot tell the percentage of participants who agreed	2 Study is designated as a cohort analytic study	3 Confounding variables were not discussed	2 Cannot tell if outcome assessors were aware of intervention status Cannot tell if intervention participants were aware of research question	1 Tools were referenced as valid and reliable	3 Cannot tell if there were withdrawals or dropouts	Weak quality: As this study scored two weak ratings, the overall judgement is weak quality
Summary of bias across the studies	Four studies were of strong quality and controlled for selection bias, the remaining 4 were of moderate quality	Three studies were of strong quality for study design, and the remaining 5 were of moderate quality	Most studies (*n* = 5) did not control or disclose information on confounders and were designed weak quality. The following three were designated as strong, with sufficient information provided	Seventy five percent of the non-randomised studies were of weak quality for blinding participants and outcome assessors. Fifteen percent were of moderate quality	Three studies were of strong quality and referenced adequate validity for outcome measures, while 5 studies did not describe validity, resulting in weak quality	There was a mixture of strong (*n* = 3), weak (*n* = 3) and moderate (*n* = 2) for the researchers disclosure of follow-up rates and dropouts	On two outcomes (selection bias and study design), the included studies were of strong or moderate quality. There was a combination of strong and weak scores for confounding variables and outcome measures and moderate and weak for blinding. Mixed findings were indicated for withdrawal rates, comprising a range of strong, moderate and weak studies

1 = strong, 2 = moderate, 3 = weak

Across the non-randomised studies, all eight scored strong or moderate for selection bias and study design methods. However, most studies scored weak for controlling for (a) confounding variables (*n* = 5/8), (b) utilising valid outcome measures (*n* = 5/8) and (c) blinding participants and outcome accessors (*n* = 6/8). Mixed findings were indicated for withdrawal rates, comprising a range of strong (*n* = 3), weak (*n* = 3) and moderate (*n* = 2) ratings.

### Outcome measure validity assessment

Five studies were deemed to have acceptable outcome measures as two [69, 74] conducted and reported adequate internal consistency, and three [37, 68, 73] referenced psychometric validity from previous studies. The remaining five studies [29, 67, 70–72] were deemed unacceptable for outcome measurement validity as they did not meet our predefined criteria. In terms of validity for specific outcomes, in six studies [29, 37, 67, 69, 71, 74], five different measures were used to assess levels of mental health knowledge, of which three [29, 67, 71] provided no evidence for validity. The three studies [37, 67, 69] assessing stigma employed three different outcome measures, and one study [67] did not provide evidence for validity. In the five studies assessing referral efficacy/confidence [29, 37, 67, 71, 74], all but one [74] used a validated scale. Help-seeking intentions and behaviour were assessed with valid outcomes in two [37, 69] studies.

## Discussion

This systematic review was a response to an increasing recognition that athletes, coaches and officials in sport settings can be vulnerable to mental health problems [12, 13, 22]. We sought to gather evidence for the potential effectiveness of mental health awareness programs for improving mental health knowledge and help-seeking among coaches, athletes and officials. Studies that met the inclusion criteria were reviewed for quality so that recommendations for those in the process of designing and evaluating studies could be made.

### Effects of studies on awareness outcomes

The review revealed positive effects on indices of mental health knowledge such as recognition of disorders and use of treatments in six interventions [29, 37, 67, 69, 71, 74]. However, all but two [69, 74] reported an effect size, limiting clinically significant interpretations. A further three [29, 67, 71] provided no evidence for the psychometric validity of their knowledge measures. Moreover, the Gulliver et al. [69] study was the only one to use randomisation procedures and include a follow-up. Therefore, given the lack of methodological rigour across the studies, little confidence can be drawn on the long-term effectiveness of sport-based mental health awareness programs on increasing knowledge.

Results revealed reductions in stigma surrounding mental health disorders in both the short [37, 67] and long-term [69]. While there was a low risk of bias and acceptable measurement validity in two of these studies [37, 69], one [67] presented a high risk of bias and did not provide evidence for instrument validity. Notwithstanding the methodological issues to be overcome, these findings suggest that training athlete role models to address stereotypes and convey de-stigmatising information may be an efficacious intervention method to reduce stigma.

While perceptions of self-efficacy to help someone with a mental health problem was enhanced in five studies [29, 37, 67, 71, 74], just one [74] reported effect sizes, used randomisation procedures, maintained the effects longitudinally, and evidenced validity for their referral efficacy instrument. As such, there is limited clinical significance and long-term evidence for the current programs on improving referral efficacy.

The review revealed that one study [37] reported an increase in intentions to offer help to those with a mental health problem in the short-term, albeit without indicating the strength of the effect. Another intervention [69] indicated a null effect for both intentions and behaviour to help oneself in the short and long term. Given actual behaviour change was not found in these two interventions, or indeed measured in the remaining eight, there is scope for future programs to explore behaviour change modalities in the field of mental health promotion in sport (see [75–78] for behaviour change frameworks).

Mental health and well-being was improved in three studies [68, 70, 73]. However, the Longshore and Sachs [70] article was the only intervention to report effect sizes wherein a clinically meaningful reduction of negative affect was revealed (*d = .*21), and a null effect was indicated for positive affect. Although this study comprised a small sample size, these findings indicate that mindfulness may be an efficacious method for enhancing well-being in coaches. Taking the review’s findings collectively, there was no evidence of any negative effects of the interventions.

### Methodological quality of studies

Close inspection of the studies indicate various design limitations, these need to be overcome for future development of programs. For example, five studies did not include a control group, and of the five that did, only two [69, 74] used randomisation procedures and reported effect sizes. Therefore, the clinical significance and long-term effectiveness of the existing programs on mental health knowledge, stigma, referral efficacy and well-being outcomes remains inconclusive.

The sample sizes included in each study were generally small and are indicative of the small number of programmes that were appraised. Two studies [71, 73] did not report on the gender figures for their sample, and therefore, prior evidence regarding a gender effect for help-seeking behaviours [32, 33] remains inconclusive in the current review.

By profiling the study quality and risk of bias using appraisal tools, only one study [69] was deemed as having a low risk of bias, one study was unclear for bias [74] and seven of the eight non-randomised studies were of weak quality. Across the studies, there were methodological concerns reported for the controlling for confounding variables and blinding of participants and outcome accessors. Mixed findings were evident for random sequence generation and withdrawal rates. In addition, five of the ten included studies did not meet our predefined criteria for acceptable psychometric measurement validity, and there was substantial heterogeneity and limited validity for the majority of referral efficacy and mental health knowledge instruments. Based on the findings, it is difficult to draw confidence in the effects reported by some of the studies included.

Researchers in this area should consider adhering to available methodological guidance for psychometric measures (i.e. face, construct, discriminant, concurrent, predictive, nomological validity assessment) (see, [79]) and design and report their interventions in line with study protocols such as those provided by the Consolidated Standards of Reporting Trials [40].

### Intervention delivery methods

The content of each of the programs varied, and the attendees involved were from a variety of backgrounds within a sport setting, i.e. elite athletes, coaches, club leaders, student athletes, officials, and those athletes who had been referred after reporting substance misuse (see Table 3). Therefore, future reviews may want to consider limiting the search to a particular group only (i.e., athletes, coaches, officials or athletes considered to be at high risk).

Similarly, the frequency and duration of sessions for each program varied from an 8-h program across three group sessions [67], 12 separate groups sessions each with a different topic, to a program that lasted 1.5 h initially then completing a home program for 6 weeks [70]. Two programs were delivered online [69, 74] while six were delivered in groups by trained facilitators. Determining the most effective delivery method (i.e., online, one to one, in groups) and intervention, duration and frequency are not possible from the current review, but could be considered a screening variable for future reviews.

### Limitations and recommendations

Individual case study work of applied sport psychologists was not incorporated within this systematic review. A potential avenue to facilitate a review of single case study reports is through journals publishing case studies and recent books including evidence-based psychological interventions in sport [80]. The current review excluded non-peer-reviewed articles, whereas a review on grey literature (e.g. programs published by government, national public health agencies, sports bodies, and mental health charitable organisations) could be considered. As previously indicated, we did not conduct meta-analyses as the operationalisation, measurement and statistical reporting of the constructs lacked consistency and methodological rigour. Therefore, the predictive and cumulative effects on the programs on mental health awareness indices (i.e. *stigma*, knowledge, referral efficacy, help-seeking) remains unclear.

It was not apparent from the current review that any of the programs selected were underpinned by behaviour change theory, or guidelines, like those proposed by the Medical Research Council (MRC) on developing and evaluating complex interventions [81]. Future programmes could consider the inclusion of these guidelines. We also make a recommendation that psychological theories such as the Self-Determination Theory [75], Theory of Planned Behaviour [76] or the Health Belief Model [77] be considered in developing and evaluating interventions. The choice of theory should be determined with the planned outcomes of the intervention in mind, and also the availability of valid and reliable measurement tools for the specific sporting populations targeted. We refer the reader to recent research on the taxonomy of behaviour change as a guide for choosing an appropriate theory [78].

## Conclusions

Evidence and theory-based intervention programs designed to increase mental health literacy and support athletes, coaches and officials who are experiencing a mental health problem are required. While some support was found for the programs available, few showed methodological quality and most suffered a high risk of bias. None of the studies followed the standards for reporting trials, referred to in the MRC guidelines or conducted long-term follow-ups (beyond 3 months). Future longitudinal studies are required with larger sample sizes of males and females, wherein randomisation to groups is blinded, and outcomes are measured with validated measurement tools. Program designers should also give due consideration to the integration of behaviour change theory in the development of programs. We conclude that a cautionary approach be taken when determining an effective program and encourage those involved in program design to consider some of the limitations raised in this article.

## Additional files

Additional file 1: PRISMA checklist. (DOC 62 kb)

Additional file 2: Full search for the psychinfo database. (DOCX 16 kb)

## Abbreviations

MRC: Medical Research Council
PRISMA: Preferred Reporting Items for Systematic Reviews and Meta-Analyses
PROSPERO: International prospective register of systematic reviews
QATSQ: Quality Assessment Tool for Quantitative Studies
SNI: Sport Northern Ireland
WHO: World Health Organisation
