# Supplementary material for: A systematic review of interventions to increase awareness of mental health and well-being in athletes, coaches and officials
Source: Syst Rev. 2017 Aug 31;6:177. doi: 10.1186/s13643-017-0568-6 (PMC5579872; doi:10.1186/s13643-017-0568-6)
Supplement: Supplementary file 2 — Full search for the psychinfo database. (DOCX 16 kb) [file 13643_2017_568_MOESM2_ESM.docx]

| Mental Health Interventions  PsychINFO  1. exp sports/  2. Sport$.ab,ti.  3. 1 or 2  4. exp teachers/  5. (Leader$ or teacher$ or instructor$ or player$ or member$ participant$ or coach$).ab,ti.  6. 4 or 5  7. 3 and 6  8. sports coaching/  9. exp athletes/  10. athlete$.ab,ti.  11. 8 or 9 or 10  12. 7 or 11  13. schools/ or colleges/ or high schools/  14. (Sport$ adj3 (organi#ation$ or club$ or governing bod$ or cent$ or school$ or setting$)).ab,ti.  15. 13 or 14  16. 12 and 15  17. mental health/  18. well being/  19. (mental$ adj3 (health or wellbeing or well being or well-being or wellness or ill$)).ab,ti.  20. anxiety/  21. "depression (emotion)"/  22. (anxiety or depress$).ab,ti.  23. 17 or 18 or 19 or 20 or 21 or 22  24. 16 and 23  25. internet/  26. websites/  27. (internet or online or website$ or web site$ or web based).af.  28. 25 or 26 or 27  29. 15 or 28 |
| --- |
